# Empowering Veterinarians to Be Planetary Health Stewards Through Policy and Practice

**DOI:** 10.3389/fvets.2022.775411

**Published:** 2022-03-03

**Authors:** Dilara Kiran, William E. Sander, Colleen Duncan

**Affiliations:** ^1^Department of Microbiology, Immunology, and Pathology, College of Veterinary Medicine and Biomedical Sciences, Colorado State University, Fort Collins, CO, United States; ^2^Department of Veterinary Clinical Medicine, College of Veterinary Medicine, University of Illinois at Urbana-Champaign, Urbana, IL, United States

**Keywords:** climate change, veterinary medicine, policy, public health, education

## Abstract

Veterinarians are established public health professionals, committing to promote public health when they take their veterinary oath. The issue of climate change and its impact on planetary health is vital to public health, and therefore, it is critical that climate change is regarded as within the veterinary scope of practice. However, climate change is a multi-faceted issue which requires interdisciplinary collaboration and integrated stakeholder involvement in order to establish effective solutions and impactful policies. As a result, in this perspective, we discuss how policy is critical to support veterinarians in the climate change space and argue that more explicit support is needed for veterinarians to take an active role in climate change adaption, resilience, and mitigation. We address the discrepancies between the human health and veterinary professions with respect to providing policy support and capacity for practitioners to be stewards to promote planetary health and shed light on the lack of veterinary capacity in this area. We stress that veterinary professional societies are well equipped to bolster their policies, expand education for veterinary professionals and students in policy and advocacy, and establish calls to action to address climate change and planetary health issues. Ultimately, as public health professionals, veterinarians are uniquely poised to be contributors to climate change solutions and they should be actively involved in policy decision-making and empowered to take active roles in interdisciplinary conversations surrounding this important issue.

## Introduction

Veterinarians are critical public health professionals. Veterinary practitioners in the United States (U.S.) and across the globe, regardless of sector, commit to promoting public health when they take their veterinary oath (1–3). The nature of veterinary training inherently promotes an interdisciplinary approach to solving global health problems, including integrated use of public health practices such as disease detection, reporting, and surveillance, as well as health education and prevention (1). It is well established that the health of animals contributes to the health of humans, and vice versa, in multiple notable areas, by protecting against zoonotic disease, bolstering a sustainable food supply, fostering the human-animal bond, and preserving ecological systems (4, 5). Involving veterinarians in decision making has significant advantages over tackling problems in health professional silos, as has been the case for coordinated human and animal vaccination efforts against zoonoses, integrated diagnostic laboratory infrastructure, and integrated surveillance and response to disease outbreaks (6). The importance of interdisciplinary collaboration, including veterinarians, to solve global health problems has been exemplified with COVID-19. The issue of climate change and its impact on planetary health is no different.

**Figure 1:**
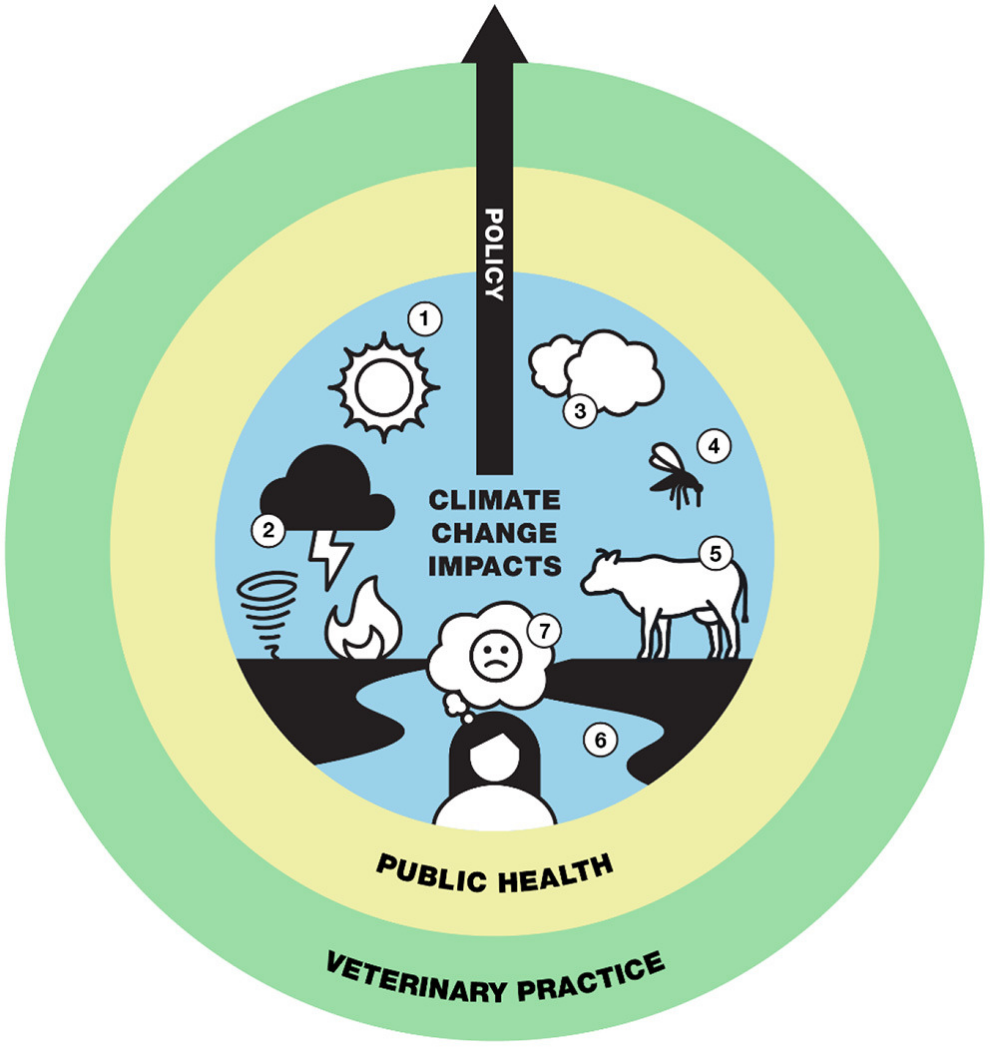
Policies already exist which highlight issues impacted by climate and which fall within a One Health and planetary health framework. These issues include (1) increasing temperature, (2) extreme weather events, (3) air quality, (4) vector-borne disease, (5) food safety/security, (6) water-related health issues, and (7) mental health. These issues are not independent and should be framed within policy as climate change issues. Further, climate change issues are within the scope of public health, which is within the scope of veterinary practice. Policy is a bridge and transcendent tool that spans all spheres and has the ability to build veterinary capacity and empower the profession to be climate change stewards and protect planetary health.

Recent commentary has stressed the need for veterinarians to acknowledge climate change as within their scope of practice, redefine their roles in the climate change space, and take ownership and active roles as stewards of climate change adaptation and resilience (7). As Figure 1 demonstrates, several aspects of climate change impact public health and veterinary practice. Ultimately, climate change is a public health problem, veterinarians are public health professionals, and, thus, climate change and the promotion of planetary health are veterinary issues. However, these kinds of activities cannot be conducted within a vacuum, and support from cross-sector collaborations, stakeholders, and policy are critical for veterinarians to be effective stewards in this area (4, 7). Gaps must be bridged between veterinary practice and policy decision-making to ensure veterinarians are supported in public health capacities. In this perspective, we discuss the importance of policy to support veterinarians in the climate space and argue that policies should more explicitly support the role of veterinarians and prepare them to take climate action.

## The Role of Policy And Why it Matters

Policy is a vital tool for all health practitioners, including veterinarians. There are two explicit ways in which science and policy interface, science for policy and policy for science (8). Clinical knowledge, expertise in global public health issues, understanding of zoonotic disease transmission, and translational research efforts using animal models are all examples of areas in which the knowledge acquired through veterinary training can be used to help inform legislative decisions that require scientific and clinical expertise (science for policy). However, policy is also generated which impacts how veterinary practice is conducted (policy for science). Policy for science shapes how drugs are dispensed, the regulation of generated waste, drug compounding, tax laws for small businesses, employee healthcare programs, loan repayment programs, the nature of a veterinary-client-patient relationship, ethical responsibilities for a veterinarian, legal definitions of animals, occupational requirements, and modalities of practice, such as telemedicine. Spanning the practitioner-policy boundary is critical for veterinary medicine, ensuring policy decisions are rooted in evidence and are relevant to everyday practice. Effective, efficient, transparent, responsive, and proportionate policies require continuous evolution and evaluation based on current contexts (4). Through education and practice, veterinarians become competent and employ skills in clinical reasoning, individual animal and population health, public health, communication, collaboration, ethics, and workplace management. As a result, veterinary practitioners are uniquely equipped to provide the breadth of on-the-ground perspectives to inform policy affecting human, animal, and environmental health, or One Health. There is a clear, recognized need to establish and build capacity to support veterinarians in the policy space.

Policy for veterinary medicine in the U.S. originates from multiple avenues, including at the federal, state, and local level. The American Veterinary Medical Association (AVMA) is the professional society whose mission includes advocating for its members to advance the science and practice of veterinary medicine including supporting stewardship and promotion of public health (9). The AVMA closely monitors all federal and state legislation currently being discussed and comes to consensus positions to support, watch, or oppose proposed legislation. The AVMA relies heavily on volunteer committees and councils to inform policy decision-making and to inform how the Government Relations Division advocates for veterinary medicine on Capitol Hill in Washington, D.C. AVMA brokers critical meetings with stakeholder organizations, including government agencies, trade organizations, industry, and professional societies to ensure that policy most accurately supports its veterinary membership. Policies created by the AVMA cover the breadth of veterinary medicine, including licensure and accreditation, euthanasia, animal welfare, and mass depopulation. Government agencies, varied stakeholders, and veterinary practitioners look to these policies for guidance, especially in uncharted territory. A recent example highlights this influence when the United States Department of Agriculture and the swine industry needed to undergo mass depopulation during the COVID-19 pandemic, demonstrating how policy coordination across organizations could be leveraged for other pertinent global issues such as climate change (10). Federal laws, such as the Animal Welfare Act and the Humane Slaughter Act, regulate standards of care, along with practice regulations set by State Boards of Veterinary Medicine. Additionally, state Veterinary Medical Associations provide specific legislative updates and expertise for community-based issues. Ultimately, professional societies have unique roles and responsibilities, which include bridging gaps between practitioners and policy and providing support and guidance on taking active roles in the policy space for relevant issues, including climate change (11).

## Where is Climate Policy to Support Veterinarians?

A brief scoping review (12) was conducted in March 2021 to better understand the policy landscape that supports veterinarians in this space. The authors conclude that policies that formally support veterinarians in the climate space and articles on the need for veterinarians to be climate change leaders are virtually nonexistent. The current AVMA Committee on Environmental Issues priorities (13) and joint statements with the Canadian Veterinary Medical Association and the Federation of Veterinarians of Europe on the role of veterinarians in protecting public and environmental health fail to mention climate change (14). The only AVMA policy mentioning climate change, called “Global climate change and One Health,” has no action guidance or capacity building for veterinarians to be leaders in this space. Rather, the policy only encourages research, education, and stakeholder collaboration (15). Recently, the World Veterinary Association published a position statement declaring global climate change as an emergency, encouraging increased collaborations amongst stakeholders, and emphasizing the importance of building veterinary capacity to prevent and address climate change concerns (16). This is the only major veterinary position statement on this issue.

## How Does Policy Support Other Healthcare Professions in the Climate Space?

The human medical profession, including specialist doctors and nurses, have established calls to action and incorporated climate change as an important issue into their professional society policies (Table 1). These groups have created coalitions and subsequent policy action agendas, excluding any representation from animal health sectors. Guides to support practitioners in climate change and its impact on human and planetary health have been published in the human medical field (Table 1). For example, The American College of Physicians has developed a Climate Change Action Plan and Toolkit, included in Table 1, which not only provides a freely accessible presentation to explain the impacts of climate change on health, but provides talking points for regional U.S. climate change issues. This resource includes fact sheets to facilitate discussions about climate change directly with patients, and guides on how to create more sustainable hospital environments. Medical professionals have acknowledged the importance of incorporating climate education into training programs (17), showcasing active movement to create change in this space and a clear recognition of the professional need. Additionally, the human medical profession views climate change and sustainability as a public health issue (18–24). This is true across physicians (25–30), nurses (31–38), and dentists (39, 40). The need for these professionals to use their voices for change, be powerful advocates, and be agents of action in this space has been emphasized (41–44). The veterinary profession can adapt human health coalition policy agendas and practitioner guides within their own organizations.

**Table 1:** Select climate change policy calls to action, resources, and practitioner guides.

**Policy resource/guide**	**Link to resource**
Climate change toolkit from the American College of Physicians	https://www.acponline.org/advocacy/advocacy-in-action/climate-change-toolkit
A physician's guide to climate change, health, and equity	https://climatehealthconnect.org/resources/physicians-guide-climate-change-health-equity/
Health voices for climate action coalition policy action agenda	https://climatehealthaction.org/cta/climate-health-equity-policy/
Canadian Association of Physicians for the Environment–climate change toolkit for health professionals	https://cape.ca/campaigns/climate-health-policy/climate-change-toolkit-for-health-professionals/
American Medical Association recognition of climate change as health emergency	https://ama.com.au/media/climate-change-health-emergency
American Medical Association policy on climate change education	https://policysearch.ama-assn.org/policyfinder/detail/climate%20change?uri=%2FAMADoc%2FHOD.xml-H-135.919.xml
International Council of Nurses position statement on nurses, climate change and health	https://www.icn.ch/sites/default/files/inline-files/ICN%20PS%20Nurses%252c%20climate%20change%20and%20health%20FINAL%20.pdf
Alliance of Nurses for Health Environments: climate change, health, and nursing, a call to action	https://www.nursingworld.org/practice-policy/work-environment/health-safety/environmental-health/

## How Can Veterinarians Be Valuable Assets For Climate Change Solutions?

As public health professionals, veterinarians should be viewed as keystone contributors to climate change solutions. The impact of heat stress on livestock health provides an illustrative example of how veterinarians can play critical roles in climate change action. Rising temperatures lead to reduced livestock productivity and immune suppression, making livestock more vulnerable to disease and impacting food security (45). Veterinarians who provide care to livestock have the ability to inform early interventions that protect animals from heat stress and services to aid in the recovery from heat events (adaptation), bolster the overall health of populations to be better equipped to handle subsequent climate change threats (resilience), and guide dietary and other management actions that reduce cattle emissions, which contribute to warming (mitigation) (45). Similar veterinary insight can be brought to other climate change issues outlined in Figure 1. Based on this, it is unclear why veterinarians are out of the conversation surrounding climate change action. Veterinarians may not think they belong in the space, may view it as a “political” issue, or may be hesitant without supportive professional society leadership. Additionally, other professions may have excluded veterinarians in discussions of climate change, maintaining silos that would benefit from interdisciplinary expertise. As an example, veterinarians are more trusted than physicians and viewed positively by the public (46), which can be beneficial in being part of these important topics. There are guides for other health professions on having conversations about politicized topics which have major impacts on patient health (47). Human health professionals have already emphasized the need to teach patients about the relevance of climate change to direct patient care (48). By utilizing guides already created, an opportunity exists for veterinarians to be educators on how climate change impacts the health of their patients, clients, and the environment. The veterinary profession should rise to meet other healthcare professions and lead in providing policy to support climate action.

At the heart of sustained interactions between policy-makers and practitioners is communication and relationships (49). Veterinarians are trained in these skills, as this type of engagement is central to successful veterinary-client-patient relationships. Veterinarians can serve as “knowledge brokers” using cross-disciplinary communication skills, coupled with their unique perspective on climate change vulnerabilities, to establish strong relationships and trust with policy-makers early (50). In this way, veterinarians can continue to be long-term stewards for issues related to climate change and planetary health. To be successful, these types of networks and information exchange between policy-makers and veterinarians must be formally supported and facilitated (50). Within the U.S., the AVMA has the opportunity to enhance policy capacity and support reframing the scope of practice to include issues related to climate change and planetary health. Policy support available to tackle climate change issues should be equivalent between human and animal health sectors. As a model for the U.S., the Health Environment Network in Europe creates and fosters collaborative, interdisciplinary health networks to solve environmental health problems and provide policy guidance (51).

## Re-Framing Veterinary Scope of Practice to Explicitly Include Climate Change

Veterinary policies are already in place that address issues that are impacted by climate (Figure 1, Supplementary Table 1). However, these policies are often not framed within climate change but rather as independent factors. There is ample literature linking medical and public health issues to climate change, including increasing global temperature (52–57), extreme weather events (58–63), air quality (64–71), vector-borne disease (72–79), food safety/security (80–87), water-related health issues (88–94), and mental health (95–102). Health concerns have also been directly attributed to climate change (103). Yet, the disconnect within policy still exists, and a critical gap needs to be addressed to provide veterinarians with the roles, responsibilities, and support to be effective climate change advocates alongside their human medical counterparts.

## What Policy Does the Veterinary Profession Need to Support Climate Change Action?

There is a clear path forward for veterinarians to take an active role in promoting planetary health in the fight to mitigate climate change impacts. Veterinarians have the opportunity to serve as “boundary-spanners” and “honest brokers”(104) using their roles as practitioners to share their knowledge about public health issues with policy-makers, educate the public on the impacts of climate change on planetary health, and bolster capacity for climate change resilience, adaptation, and mitigation. The role of veterinarians in climate change must be recognized as critical by professional societies, such as the AVMA. Programs that allow veterinarians to gain experience at the science-policy interface, such as the AVMA Fellowship Program, must be strengthened. Specific training opportunities, such as fellowships in climate change and health policy and creating climate-specific advocacy toolkits and practitioner guides, would empower veterinarians to act (105–108). Professional society leadership are well poised to develop and teach these skills to practitioners (105, 106). More effort is needed from veterinary professional societies to publish calls to action and take an overt stand that puts veterinarians in the forefront of climate change efforts. While some advocacy programming exists at both state and federal levels, they are limited in their breadth and depth. Creating continuing education modules for advocacy and policy is one way to enhance this knowledge among veterinarians. The AVMA could take a more active role in surveying practitioners about how policy directly impacts their practice, to better emphasize the role policy plays in shaping their ability to promote public health (109). Veterinarians can look to other scientific societies that are leaders in the human health and scientific space, including the Union of Concerned Scientists and the American Association for the Advancement of Science, which have ample tools, resources, and training available for scientists to effectively navigate the policy space.

## What Educational Support Does the Veterinary Profession Need to Be Prepared Climate Change Stewards?

The desire to cross-train health professionals in policy, including knowledge of the legislative process, policy-writing, and understanding how policy shapes global topics of concern, is not new, and has been undertaken by some university programs (110). However, these types of opportunities are limited within the U.S., particularly for current veterinarians, and are not incorporated as core curricular programs. While not all veterinarians need to have explicit roles in the policy space, all should understand how their work fits into the broader, global context to promote planetary health and take action to inform policy-decision making in their area. Courses to engage current veterinary students in public policy topics that are already established (110) should be expanded as core curricula or be incorporated and integrated into already existing coursework. Integrating climate and health education is critical for fostering future generations of informed healthcare leaders (111). Veterinarians should be trained in global perspectives to allow them to be stewards for global problems, such as preserving planetary health and mitigating climate change (112). Empowering veterinarians and veterinary students to envision how policy fits into their careers and utilize their expertise to impact policy decisions from the local to international level, regardless of their chosen specialty, is critical. Public health education already views knowledge about policy as a core educational domain and competency (113). The AVMA Council on Education and the American Association of Veterinary Medical Colleges should enhance public health related professional competencies to encompass this aspect of knowledge and make a commitment to including public policy and the practitioner-policy interface as key skillsets.

## Concluding Remarks

Policy to support veterinarians as climate change leaders will not only benefit veterinarians but will contribute to the overall effort to improve planetary health. The next generation of veterinarians, veterinary technicians, and clients all care about the changing climate. To stay socially relevant as veterinarians, it is critical for the veterinary profession to address climate change as a need (7). Veterinary students across the U.S. agree that climate change is a problem, believe veterinarians have a responsibility to be leaders in this space, that veterinary professional societies should take an active role, and that educational opportunities should be expanded (114). Veterinary medicine should re-consider its curricular foci and priorities to incorporate policy and advocacy at the forefront of translatable skillsets. The importance of incorporating project-based, experiential learning opportunities that model real-world, complex problems has been demonstrated for the related area of sustainability education (115). This type of applied learning helps prepare students to be change leaders and ensures that they have the capacity to translate curricular knowledge into practice and policy settings. Federal agencies, senior leaders in higher education administration, program directors, department faculty, and private sector organizations all have roles to play in fostering the development of core competencies, researching curricular effectiveness, encouraging interdisciplinary program building, using problem-based learning approaches, enhancing mentorship and advising capacity, and promoting cross-sector collaborations to build a comprehensive educational program in climate change and policy (115).

The call to action from health professions is clear. A pledge has been proposed to incorporate the protection of planetary health, including mitigating climate change, into the ethos and framework of healthcare professions and to incorporate planetary health into educational offerings (17, 116). This call to action should include the veterinary profession. Veterinarians must be empowered to protect planetary health and have a sense of ownership and stewardship about protecting the planet against the impacts of climate change. In order to do this, veterinarians need to be supported in a professional capacity to do work in this space, and policy is a critical piece of this puzzle. Climate change policy is relevant to all veterinary practitioners, whether they work in clinical practice settings or elsewhere, because climate change is a public health issue (Figure 1). Advocacy skills are key, and veterinarians can be involved with the policy-making process and build these skills at all levels, from the local level via county public health boards to the state and national levels through professional societies and relationships with policy-makers. Veterinarians can serve as critical community leaders to advocate for concerns relating to climate change and health of their patients and clients (117).

Ultimately, when veterinarians take their veterinary medical oath, they vow to promote and protect public health, and medical professionals agree that climate change is currently the greatest public health threat (118). If veterinarians want to maintain their commitment to promoting public health, they must be able to address climate change (119), be actively included in conversations as key stakeholders for policy decision-making, and be formally supported and trained in public policy and advocacy.

## Data Availability Statement

The original contributions presented in the study are included in the article/Supplementary Material, further inquiries can be directed to the corresponding author.

## Author Contributions

DK and CD initiated the study. DK conducted the scoping review, organized resources, wrote the first draft of the manuscript, and designed the initial figure. WS, CD, and DK reviewed results and contributed to interpretation. All authors contributed to manuscript revision, read, and approved the submitted version.

## Conflict of Interest

The authors declare that the research was conducted in the absence of any commercial or financial relationships that could be construed as a potential conflictof interest.

## Publisher's Note

All claims expressed in this article are solely those of the authors and do not necessarily represent those of their affiliated organizations, or those of the publisher, the editors and the reviewers. Any product that may be evaluated in this article, or claim that may be made by its manufacturer, is not guaranteed or endorsed by the publisher.
