# Supplementary material for: Empowering Veterinarians to Be Planetary Health Stewards Through Policy and Practice
Source: Front Vet Sci. 2022 Mar 3;9:775411. doi: 10.3389/fvets.2022.775411 (PMC8928474; doi:10.3389/fvets.2022.775411)
Supplement: Supplementary file 1 [file Data_Sheet_1.PDF]

Supplementary Table 1: Climate Change Issues and Select Policies Relevant to Veterinarians

Climate Change Issue	Relevant Policies
Increasing temperature	1. AVMA policy "Transport, Sale Yard Practices, and Humane Slaughter of Hoofstock and Poultry" → "animals must be protected from environmental extremes, such as excessive heat and cold" https://www.avma.org/resources-tools/avma-policies/transport-sale-yard-practices-and-humane-slaughter-hoofstock-and-poultry<br>2. AVMA Policy "Companion Animal Care Guidelines" → "When climatic conditions pose a threat to the animal's health or well-being, taking into consideration its age, breed, overall health status, and acclimation, then appropriate measures must be taken to alleviate the impact of those conditions" https://www.avma.org/resources-tools/avma-policies/companion-animal-care-guidelines<br>3. AVMA policy "Acclimation certificates/statements" → about the transport of pet animals on airlines. https://www.avma.org/resources-tools/avma-policies/acclimation-certificatesstatements<br>4. Animal Welfare Act → standards and certification process for humane handling, care, treatment, and transportation of animals "shelter from extremes of weather and temperatures" https://www.govinfo.gov/content/pkg/USCODE-2015-title7/html/USCODE-2015-title7-chap54.htm<br>5. OIE Animal Welfare Standards → freedom from heat stress is one of the freedoms listed; https://www.oie.int/en/animal-welfare/an-international-network-of-expertise/#A<br>6. Warm Weather pet safety information: https://www.avma.org/resources/pet-owners/petcare/warm-weather-pet-safety
Extreme weather events	1. Animal Welfare Act → standards and certification process for humane handling, care, treatment, and transportation of animals "shelter from extremes of weather and temperatures" https://www.govinfo.gov/content/pkg/USCODE-2015-title7/html/USCODE-2015-title7-chap54.htm<br>2. AVMA CDEI Emergency Management Roadmap: https://www.avma.org/resources-tools/avma-policies/cdei-emergency-management-roadmap<br>3. OIE Disaster Management plan: https://www.oie.int/fileadmin/Home/eng/Animal_Welfare/docs/pdf/Others/Disastermanagement-ANG.pdf<br>4. AVMA policy on role of veterinarians in national/state/local emergencies https://www.avma.org/resources-tools/avma-policies/role-veterinary-profession-national-state-and-local-emergencies<br>5. AVMA support of an integrated animal emergency preparedness and response program https://www.avma.org/resources-tools/avma-policies/integrated-animal-emergency-preparedness-and-response-program<br>6. AVMA information about planning for natural disasters: https://www.avma.org/resources/pet-owners/emergencycare/pets-and-disasters

	7. FEMA information for pet owners: https://www.fema.gov/media-library-data/1390846777239-dc08e309debe561d866b05ac84daf1ee/pets_2014.pdf<br>8. FEMA presentation → outlines current laws about response to natural disasters/emergencies with respect to pets, and lists allied organizations, including vet med https://www.fema.gov/pdf/conferences/iaconference/2010/wednesday_830am_household_pets_intro_1.pdf<br>9. Pandemic and All-Hazards Preparedness and Advancing Innovation Act of 2019 → public health workforce capacity, reauthorizing EIS which includes veterinarians<br>10. Public Health Security and Bioterrorism Preparedness and response Act of 2002 → develop and implement coordinated public health strategies
Air quality	1. AVMA Policy "Companion Animal Care Guidelines" → "Proper ventilation removes heat, dampness, odor, airborne microbes, and pollutant gases...while allowing for the introduction of fresh air" https://www.avma.org/resources-tools/avma-policies/companion-animal-care-guidelines<br>2. Animal Welfare Act → standards and certification process for humane handling, care, treatment, and transportation of animals "ventilation" https://www.govinfo.gov/content/pkg/USCODE-2015-title7/html/USCODE-2015-title7-chap54.htm<br>3. AVMA Policy "Layer Hen Housing Systems" → must provide...air quality...that promote good health and welfare" https://www.avma.org/resources-tools/avma-policies/layer-hen-housing-systems<br>4. AVMA Policy "Pregnant Sow Housing" → promote good air quality https://www.avma.org/resources-tools/avma-policies/pregnant-sow-housing<br>5. Agriculture/Air Quality – Clean Air Act related → https://www.epa.gov/agriculture/agriculture-and-air-quality; air monitoring at agricultural operations : https://www.epa.gov/afos-air<br>6. USDA and EPA memorandum of understanding about air quality → https://www.epa.gov/sites/production/files/2016-06/documents/usda_epa_mou_1998.pdf<br>7. Veterinary Compliance Assistance site about Clean Air Act → https://www.vetca.org/regsandstandards/caa.php<br>8. USDA Natural Resources Conservation Service info about Air Quality https://www.nrcs.usda.gov/wps/portal/nrcs/detail/national/air/quality/?cid=stelprdb1047129
Vector borne disease	1. AVMA policy "Animal disease control program supervision" → all animal disease control and eradication programs should be under veterinary supervision https://www.avma.org/resources-tools/avma-policies/animal-disease-control-program-supervision<br>2. AVMA policy "Preventing entry of foreign animal diseases (transboundary diseases), their vectors, and invasive species" → about supporting surveillance and control measures https://www.avma.org/resources-tools/avma-policies/preventing-entry-foreign-animal-diseases-transboundary-diseases-their-vectors-and-invasive<br>3. AVMA Policy → Zoonotic Disease Education; education programs should include content about ways zoonotic disease are transmitted, including vector, and biosecurity, epidemiology, outbreak response, etc.

	https://www.avma.org/resources-tools/avma-policies/zoonotic-disease-education<br>4. OIE Terrestrial Animal and Aquatic Health Codes; also Diagnostic and Vaccine Manuals → standards for animal health/welfare and veterinary public health related to trade https://www.oie.int/en/standard-setting/overview/<br>5. OIE Biothreat Reduction Strategy https://www.oie.int/en/scientific-expertise/biological-threat-reduction/
Food safety/security	1. AVMA policy "Food animal health emergency planning" → protection from bio/agro terrorism and natural disaster threats https://www.avma.org/resources-tools/avma-policies/food-animal-health-emergency-planning<br>2. AVMA policy "Food Safety" → "the veterinary profession is the only health profession that is actively involved in all aspects of the food chain from farm production of food animals to the consumption of food products" https://www.avma.org/resources-tools/avma-policies/food-safety<br>3. AVMA Policy "Processes for microbial reduction in food" → AVMA support of pasteurization, irradiation, etc. https://www.avma.org/resources-tools/avma-policies/processes-microbial-reduction-food<br>4. AVMA policy "Raw milk" → opposing direct sale or distribution of unpasteurized milk/dairy products https://www.avma.org/resources-tools/avma-policies/raw-milk<br>5. AVMA policy on GMO and GE foods https://www.avma.org/resources-tools/avma-policies/safety-genetically-modified-organisms-gmo-and-genetically-engineered-ge-foods<br>6. AVMA policy "The importance of Veterinarians in Food Safety" → support of USDA FSIS workforce – all slaughter plants must be under the direct supervision of a veterinarian https://www.avma.org/resources-tools/avma-policies/importance-veterinarians-food-safety<br>7. AVMA policy on food labeling https://www.avma.org/resources-tools/avma-policies/truthful-and-nonmisleading-human-food-labeling<br>8. AVMA policy on expanding veterinary student training in food safety, security, defense https://www.avma.org/resources-tools/avma-policies/veterinary-student-training-programs-food-safety-security-and-defense<br>9. AVMA policy on global food security → https://www.avma.org/resources-tools/avma-policies/global-food-security<br>10. OIE International Standards https://www.oie.int/en/food-safety/oie-activities/; general session resolutions related to animal production food safety → https://www.oie.int/en/food-safety/animal-production-food-safety/resolutions-on-apfs/<br>11. Veterinary shortage areas – including food safety, National Veterinary Medical Service Act provides loan repayment to those who wish to serve there<br>12. FDA Food Safety Modernization Act – Center of Veterinary Medicine increasing staff, improving food inspection/importation<br>13. Securing our Agriculture and Food Act → coordinate DHS efforts related to defending food, agriculture, and veterinary systems; veterinary public health activities
Water related health issues	1. AVMA Best Management Practices for Pharmaceutical Disposal → never four or flush pharmaceuticals down drains or toilets

	https://www.avma.org/resources-tools/avma-policies/best-management-practices-pharmaceutical-disposal<br>2. AVMA Guidelines for Addressing Hazards in the Workplace → must comply with OSHA regulations https://www.avma.org/resources-tools/avma-policies/guidelines-addressing-hazards-workplace<br>3. European focus, but policy highlights for pharmaceutical residues in freshwater from the Organization for Economic Cooperation and Development – international organization, United States is a member organization https://www.oecd.org/environment/resources/pharmaceutical-residues-in-freshwater-policy-highlights.pdf<br>4. EPA Regulations on Animal Feeding Operations; regulatory compliance under the Clean Water Act, National Pollutant Discharge Elimination System → https://www.epa.gov/npdes/animal-feeding-operations-regulations-and-guidance<br>5. AVMA has various policies on judicious use of antimicrobials – include environmental contamination via water medicators/feeders<br>6. Veterinary Compliance Assistance site about Clean Water Act → https://www.vetca.org/regsandstandards/cwa.php
Mental health	1. AVMA policy on Harassment and discrimination-free workplaces https://www.avma.org/resources-tools/avma-policies/harassment-and-discrimination-free-veterinary-workplace<br>2. AVMA policy on diversity and inclusion https://www.avma.org/resources-tools/avma-policies/avma-policy-diversity-and-inclusion<br>3. Royal College of Veterinary Surgeons – Mind Matters and anti-stigma campaigns, Guide to Enhancing Wellbeing and Managing Work Stress in the Veterinary Workplace; released joint statement with AVMA about Mental Health and Wellbeing https://www.avma.org/javma-news/2018-10-15/taking-mental-health-positive-direction; http://www.vetmindmatters.org/wp-content/uploads/2018/01/MMI-12pp-web.pdf<br>4. Get Help page – resources from AVMA: https://www.avma.org/resources-tools/wellbeing/get-help<br>5. State by State Wellbeing programs for veterinarians→ include regulations about impaired practitioners, voluntary treatment, peer assistance programs etc.: https://www.avma.org/advocacy/state-local-issues/state-wellbeing-programs-veterinary-professionals
General One Health/ Environment Policies	1. CEI Roadmap for Environmental Leadership Priorities → AVMA Committee on Environmental Issue. Four environmental areas of focus: environmental health issues (no mention of climate change, but emerging disease, zoonotic disease, environmental contaminants mentioned), green practices in vet med, wastes generated by animals/veterinarians, and One Health: https://www.avma.org/resources-tools/avma-policies/cei-roadmap-environmental-leadership-priorities<br>2. Policy under review at AVMA → Joint AVMA-FVE-CVMA statement on the essential role of veterinarians in protecting animal, human, public, and environmental health – a global public good (no mention of climate change) https://www.avma.org/resources-tools/avma-policies/joint-avma-fve-cvma-statement-essential-role-veterinarians-protecting-animal-human-public<br>3. AVMA One Health Policy → one statement that says it supports advancements and awareness of One Health https://www.avma.org/resources-tools/avma-policies/one-health

	4. AVMA policy “Environmental Responsibility is a One Health Issue” Does actually mention long-term planning and promotion of science-based, environmentally sensitive practices to ensure viable ecosystem health and mitigate the impact of global climate change for future generationshttps://www.avma.org/resources-tools/avma-policies/environmental-responsibility-one-health-issue<br>5. AVMA Policy -- Global Climate Change and One Health → encourages research and education about the impacts of climate change, supports stakeholder coordination/collaboration https://www.avma.org/resources-tools/avma-policies/global-climate-change-and-one-health<br>6. AVMA Principles of Veterinary Medical Ethics → veterinary responsibility to contribute to the improvement of the community and betterment of public health; veterinarian has responsibility to seek changes to laws and regulations which are contrary to best interest of public health, responsibilities extend beyond individual patients and clients https://www.avma.org/resources-tools/avma-policies/principles-veterinary-medical-ethics-avma<br>7. OIE One Health information, incorporated into their Intergovernmental Standards https://www.oie.int/en/for-the-media/onehealth/
